# Hylacross Hyaluronic Acid Injectables in the Lips: Global Expert Perspectives for Achieving Optimal Esthetic Outcomes

**DOI:** 10.1111/jocd.71013

**Published:** 2026-07-03

**Authors:** Sarah G. Boxley, Kathleen M. Morno, Lina María Cadena Ortiz, William McGillivray, Hani Aljohani, Dario Bertossi, Carola de la Guardia

**Affiliations:** ^1^ SkinBox Fremantle Western Australia Australia; ^2^ RL Center for Cosmetic Surgery and Medspa Vernon Hills Illinois USA; ^3^ Clínica Estética Natural Imagen Bucaramanga Colombia; ^4^ Project Skin MD Vancouver British Columbia Canada; ^5^ American Aesthetic Center & Delara Surgical Day Care Aesthetic Center Riyadh Saudi Arabia; ^6^ Department of Maxillo Facial Surgery, Head & Neck Department Universita Degli Studi di Verona Verona Italy; ^7^ Global Aesthetics Medical Affairs Allergan Aesthetics, an AbbVie Company Madrid Spain

**Keywords:** filler, hyaluronic acid, Hylacross, Juvéderm ULTRA, lip augmentation, lip enhancement

## Abstract

**Background:**

Lips are a common area of concern for patients seeking facial esthetic treatment. Hyaluronic acid (HA) injectables are the gold standard for nonsurgical lip improvement, and the Hylacross products may be particularly well suited to this area.

**Objectives:**

To provide expert considerations on how Hylacross products can be used to achieve effective, individualized, natural, and durable lip outcomes in contemporary practice.

**Methods:**

An international group of clinicians with various specialties and extensive experience of these products completed a written questionnaire. Their collective clinical expertise forms the basis of the current considerations.

**Results:**

Hylacross products can be used to address a wide range of patient needs in the lips, such as counteracting age‐related changes, volume augmentation, improving symmetry and balance, border definition, and contouring. Most patients prioritize natural results. Achieving this requires adherence to key technical principles, including: whole‐face consideration; respect for individual lip anatomy and proportions; appropriate selection of product, injection device, and injection depth; and the use of small volumes within a conservative initial approach in treatment‐naïve patients. A key safety principle relates to assessment of the position and depth of the labial arteries and the use of techniques that minimize the risk of vascular compromise. Patients with significant anxiety around unnatural results may be reassured through a comprehensive approach that combines relationship building, patient education, and cautious treatment planning.

**Conclusions:**

By following these principles, Hylacross HA injectables can deliver well‐tolerated, effective, and durable outcomes in the lips.

## 1 Introduction

The lips and perioral area play key roles in social interaction, expression of emotion, age perception, and perceived attractiveness [1, 2, 3]. Lips are therefore a common area of concern for patients seeking facial esthetic treatment. Among nonsurgical modalities, injectable hyaluronic acid (HA) gels are a key option and offer a range of potential advantages, including immediate results, minimal downtime, biocompatibility, biodegradability, reversibility with hyaluronidase, and an established safety profile [4, 5].

The Hylacross range of HA injectables (Allergan Aesthetics, an AbbVie company, Pringy, Annecy, France) has been a mainstay of nonsurgical lip improvement for 25 years, since the first product was introduced to the market in 2000. The underlying technology is based on high‐molecular‐weight HA at a concentration of 24 mg/mL crosslinked with 1,4‐butanediol diglycidyl ether [6]. Formulations are summarized in Table 1. They are available under different brand names in different countries but Juvéderm ULTRA 2 is clinically equivalent to Juvéderm ULTRA XC (HYC‐24L), and Juvéderm ULTRA 3 and Juvéderm ULTRA Smile are clinically equivalent to Juvéderm ULTRA Plus XC (HYC‐24L+). Where there are differences among formulations, they vary primarily according to the degree of crosslinking, which affects their rheological and physicochemical characteristics (e.g., elasticity, cohesivity, potential water uptake). Specific indications differ between countries but each can potentially be used for lip definition and/or enhancement. It should also be noted that some Hylacross products include lidocaine and others do not, but this has no impact on effectiveness [7, 8].

**TABLE 1:** Hylacross product range.

Product	HA (mg/mL)	G'_5Hz_ (Pa)	Cohesivity/Fn (gmf)	Maximum water uptake, %
HYC‐24 (Juvéderm ULTRA)	24	156	96	580
HYC‐24L (Juvéderm ULTRA XC)	24	207	96	622
HYC‐24+ (Juvéderm ULTRA Plus)	24	214	116	515
HYC‐24L+ (Juvéderm ULTRA Plus XC)	24	263	112	454
HYC‐24L (Juvéderm ULTRA 2)	24	188	95	574
HYC‐24L (Juvéderm ULTRA 4)	24	164	105	614
HYC‐24L+ (Juvéderm ULTRA 3/Smile)	24	238	104	426

*Note:* Juvéderm ULTRA 2 and Juvéderm ULTRA XC can be considered as equivalent, as can Juvéderm ULTRA 3, Juvéderm ULTRA Smile, and Juvéderm ULTRA Plus XC. Rheologic and physicochemical characteristics are derived from de la Guardia et al. [5].

Abbreviation: HA, hyaluronic acid.

Several studies have assessed the Hylacross range of HA injectables for treatment of the lips and perioral area (Table 2) [9, 10, 11]. In the most recent of these, which supported US Food and Drug Administration approval, 213 patients were randomized to treatment with HYC‐24L (Juvéderm ULTRA XC; *n* = 157) or to a concurrent ‘no treatment’ control group in whom planned injections were deferred for ≥ 3 months (*n* = 56) [11]. Injections were made into the vermilion body and borders, as well as the perioral area (e.g., Cupid's bow, philtral columns, perioral rhytids, and/or oral commissures). The primary endpoint was met, with 79.1% of the treatment group showing a ≥ 1 grade improvement in lip fullness at 3 months (assessed using the validated 5‐point Allergan Lip Fullness Scale) compared with 26.1% of controls (*p* < 0.0001). Most subjects maintained their response out to 12 months, and the majority also reported improvements in the look and feel of their mouth. The treatment was well tolerated [11].

**TABLE 2:** Key clinical data with Hylacross products in the lips.

Study	Population size	Treatment(s)	Efficacy outcomes	Safety outcomes
Prospective, multicenter, single‐arm study [9]	*N* = 57	HYC‐24L+ (Juvéderm ULTRA Smile)	95% of investigators found the gel *very easy* to inject 99% of investigators reported improved esthetic effect vs. baseline 100% of patients reported improved lip appearance and smoothness	The treatment was well tolerated with no AEs reported
Prospective, multicenter, single‐arm study [10]	*N* = 50	HYC‐24 (Juvéderm ULTRA)	At week 12, 71% of subjects had achieved their goals for lip fullness *and* maintained improvement of ≥ 1 grade on LFS Satisfaction and goal achievement was > 90% for subjects and investigators	AEs were mostly mild– moderate in intensity and typically resolved within 1 week
Prospective, multicenter, randomized controlled trial [11]	*N* = 213	HYC‐24L (Juvéderm Ultra XC) (*n* = 157) or concurrent ‘no treatment’ control (*n* = 56)^a^	Response rate (≥ 1 grade improvement on LFS) at 3 months: treatment group, 79.1%; control, 26.1% (*p* < 0.0001) 56.4% maintained treatment response for 12 months	AEs were mostly mild– moderate in intensity One serious TRAE (angioedema at the injection site), which resolved without sequelae^b^

*Note:* Juvéderm ULTRA 2 and Juvéderm ULTRA XC can be considered as equivalent, as can Juvéderm ULTRA 3, Juvéderm ULTRA Smile, and Juvéderm ULTRA Plus XC.

Abbreviations: AE, adverse event; LFS, lip fullness scale; TRAE, treatment‐related adverse event.

^a^ This group received delayed injections at ≥ 3 months.

^b^ The subject was treated with an antihistamine and hyaluronidase injections along with a 6‐day course of prednisone.

In the intervening years since these studies were performed, the usage of Hylacross injectables in the lips has continued to develop in line with the evolution of clinical practice. The aim of the present paper is to provide expert considerations on how these products can be used to achieve effective, individualized, durable, and natural lip outcomes within the context of contemporary practice. The focus is specifically on the vermilion and its border, and not on the wider perioral area.

## 2 Methods

In September 2025, the six authors who are current esthetic practitioners independently completed a written questionnaire on their clinical experiences of lip treatment with the Hylacross portfolio of products. A blank version is available in the Data S1. The questionnaire was developed by the sponsor and covered patient profiles; treatment strategies and techniques; considerations on natural outcomes; risk minimization; and patient management. The present paper collates the joint experience of these experts, representing diverse specialties (esthetic medicine, dermatology, maxillofacial surgery, and oculoplastic surgery) from across six different countries around the world.

At the time of completing the questionnaire, all had been using one or more Hylacross products in their clinical practice for at least 13 years (range: 13–21 years). Collectively, the authors estimate that they have injected the lips of more than 12 000 patients with these products, of whom at least 5000 have been treated repeatedly (across three or more separate sessions).

### 2.1 Lip Anatomy

The lips are complex and multifunctional structures incorporating mucosal membrane, vermilion, and cutaneous surfaces [12]. The focus of the current paper is on the vermilion, but it is nonetheless important to appreciate that the superior margin of the perioral area may be considered to extend from the nasolabial folds up to the base of the nose, while the inferior margin incorporates the area between the lateral commissures and the labiomental crease of the chin [12]. The main blood supply is derived from the facial artery, which gives rise to the superior and inferior labial arteries. The key muscle is the circumferential orbicularis oris, which functions as a sphincter and (unlike most other muscles) has no direct connections to bone but rather is attached to other muscles [12]. A recent cadaver study underscored the highly compartmentalized structure of the lips [13]; six anterior and six posterior compartments were identified in each lip (thus 24 in total), with boundaries formed by vertically oriented septations.

The process of aging of the lips may be considered as a redistribution from ‘thickness’ to ‘length’, manifesting in increased prolabial length, decreased pout, vermilion inversion, and general deflation and ptosis [14, 15]. However, lip treatment is widely sought regardless of age, and patients frequently have concerns around the volume, symmetry, balance, contour, and hydration of their lips.

### 2.2 Treatment Overview

For nonsurgical esthetic lip improvement, HA injectables have long been the gold standard. Importantly, they can be used across each of the different situations described in the previous paragraph. There are other treatment modalities that can be beneficial, including botulinum neurotoxin type A (BoNT/A), platelet‐rich plasma (PRP), or energy‐based devices (EBDs). However, for optimal results, these are often best used adjunctively alongside HA injectables, rather than as an alternative. For example, BoNT/A is used off‐label for injection into the superior vermilion border and oral commissures [16], reducing the action of the superior orbicularis oris muscle—and thus leading to vermilion eversion and an appearance of greater fullness [16, 17]. This “lip flip” procedure is becoming increasingly popular for subtle contouring but does not increase actual lip volume and cannot produce the range of outcomes possible with HA‐based treatments. PRP injections can be used to stimulate collagen production and improve skin thickness, elasticity, fine lines, and texture [18, 19], but they have not been widely studied in the lip vermilion. However, there is preliminary evidence suggesting that PRP may improve lip coloration [18]. EBDs like laser and radiofrequency microneedling are also commonly used to treat the lips and have been associated with potential benefits [20, 21, 22]. For example, recent small‐scale studies have suggested that laser treatments can improve lip volume, color, and texture [21, 22]. Further studies are warranted.

### 2.3 Patient Profiles and Treatment Goals With HA Injectables

New patients presenting for the first time frequently have concerns about their lips, and this area is a common entry route into HA injectable treatment in general. The great majority of such patients are female, and it is uncommon for the lips to be a priority in males [23, 24]. However, men do sometimes request lip treatment and they were included in key studies of the Hylacross range [9, 10, 11].

Among females, there are certain profiles that commonly present seeking lip enhancement. With middle‐aged and older women, typical requests relate to countering age‐related deficits, such as general deflation and inversion. For younger women, their needs primarily relate to subtly increasing volume, enhancing balance and symmetry across the upper and lower lips, improving border definition, and/or decreasing dryness. HA injectables can be used to address each of these different issues [25, 26, 27].

Regardless of specific concerns, most patients want results that are personalized (i.e., complement their own unique facial structure), youthful, and natural. Although there has been a trend for exaggeratedly enlarged lips in some populations [28], this fashion is now waning and the great majority of patients prefer augmentation within the bounds of their natural lip shape.

### 2.4 Hylacross Injectables in the Lips

The HA products typically recommended for lip enhancement have low‐to‐medium elastic modulus (G') to provide smoothness and moldability, and medium‐to‐high cohesivity for volumization and projection [5]. The rheological and physicochemical properties of the Hylacross range largely align with these specifications, thus making them particularly well suited to lip enhancement.

For each of the clinicians in the present author group, key reasons for favoring Hylacross in the lips are provided in Table 3. Importantly, they are easy to inject, which allows for controlled administration. An ergonomically designed syringe is provided with all of the products, which facilitates the procedure (allowing better gauging of administration volume, greater injector comfort, and easier aspiration compared with its predecessor) [29]. In addition, the gels are highly moldable, thus facilitating smooth adapting to the desired shape within the treatment area. In a prospective study of lip treatment with HYC‐24L+ (Juvéderm ULTRA Smile), 95% of investigators said it was *very easy* to inject and 82% found it *very easy* to massage/sculpt [9]. The products also have a smooth consistency, which is particularly important in the lips for avoiding lumps and irregularities. In addition, they are sufficiently cohesive to provide structure and subtle volume, and to facilitate enhanced projection when needed. Importantly, the products integrate well into the tissue, which may help to ensure a natural result. Furthermore, in the authors' experience, their inherent hydrophilicity means that patients often achieve a hydrating effect, particularly when injected more superficially. All of this means that the Hylacross range of products is highly versatile and can be used at different injection depths to create different effects. Importantly, these effects are durable, with lip augmentation shown to last through 12 months (final evaluation) in a randomized trial of HYC‐24L (Juvéderm ULTRA XC) [11]. Finally, the products have been on the market for many years and thus have well‐delineated safety profiles in routine practice, and they have been shown to be both safe and effective when used appropriately.

**TABLE 3:** Reasons for using Hylacross injectables in the lips.

Authors					
HA	DB	SGB	LMCO	WM	KMM
Ease of injection Moldability Safety profile Company/product reputation	Hydrophilicity Product versatility Structural/volumizing capacity Duration of effects	Cohesivity Hydrophilicity Product versatility Duration of effects Safety/minimal swelling	Tissue integration Product versatility Structural/volumizing capacity Duration of effects	Ease of injection Smooth consistency Structural/volumizing capacity Duration of effects Safety profile	Smooth consistency Moldability Reversibility Structural/volumizing capacity Duration of effects

*Note:* Authors are listed here in alphabetical order. They were asked to provide at least three reasons why they use Hylacross products for esthetic treatment of the lips.

Within the Hylacross portfolio of products, the individual formulations used by each of the current author group are listed in Table 4. Specific indications differ among countries but each can be effectively utilized in daily practice. The decision around which one(s) to use therefore primarily comes down to the desired effects and the specific products best matched to delivering these (e.g., based on the rheological properties described in Table 1). In general, formulations within the range with lower G' and cohesivity, such as HYC‐24L, may be particularly suited to adding fine definition, whereas the other, more cohesive formulations may be best used for augmentation and structural improvement (Table 4).

**TABLE 4:** Hylacross product usage and lip techniques.

Authors	Hylacross product(s)	Techniques
HA	Mainly HYC‐24L (Juvéderm ULTRA 4) Sometimes HYC‐24L+ (Juvéderm ULTRA 3), for example, for very thin lips, delicate lip tissue, particularly subtle corrections, or when the patient is fearful of overfilling	In the vermilion, contour definition targeting the superficial dermis by linear threading (0.1–0.2 mL per side) For volume and projection in the central part of the lip body, targeting the mid‐to‐deep dermis, mainly by serial fanning (~0.3–0.4 mL in total) In the oral commissures for subtle lift, targeting the deep dermis using small boluses with 0.1 mL at each corner For restoring the peaks in Cupid's bow, targeting the superficial dermis with micro‐aliquots (0.05 mL each side)
DB	Mainly HYC‐24L+ (Juvéderm ULTRA 3) Sometimes HYC‐24L+ (Juvéderm ULTRA 4), for example, for vermilion border definition or shaping of the vermilion body	For border definition, linear retroinjection in the subdermal plane, leaving microdeposits of ≤ 0.01–0.03 mL per line For eversion and projection, vertical linear retroinjection with micro‐aliquots (0.01–0.03 mL), spaced ~1–2 mm apart, occasionally reinforcing the entry and exit points with small deposits for additional structure In the vermilion body and tubercles, submucosal injections using vertical microboluses (0.03 mL) or boluses (≤ 0.05 mL)
SGB	Mainly HYC‐24L (Juvéderm ULTRA XC) Sometimes HYC‐24L+ (Juvéderm ULTRA Plus XC) for additional lower lip projection or oral commissure support	For hydration, multiple thin horizontal threads in the subdermal dry vermilion (usually ~0.1 mL per quadrant) For tubercle enhancement, a small bolus in the subdermal dry vermilion (~0.05–0.1 mL) For projection of the lower lip, vertical S‐shaped thread around orbicularis oris or horizontal linear thread between the mucosa and orbicularis oris (0.1–0.2 mL per side) For eversion, multiple vertical thin threads in the superficial dry vermilion, from inside the vermilion border (≤ 0.1 mL per quadrant)
LMCO	Mainly HYC‐24L+ (Juvéderm ULTRA Plus XC), particularly when wanting substantial volumization and structured projection	For definition and structural support of lip contour, injection at the junction between the vermilion border and cutaneous lip using horizontal retroinjection lines within the dermal or subdermal plane (~0.02–0.03 mL per line; ~0.05 mL per area) For enhanced projection, injection within the vermilion body using vertical retroinjection lines of ~0.02 mL each, spaced 1–2 mm apart, in the subcutaneous plane or superficial fat compartment; in selected cases, entry and exit points are reinforced with small additional deposits to improve structural support (respecting anatomical boundaries to avoid lateral overfilling, migration, or disproportion) For volumization focusing on the lip tubercles, placement of microboluses or boluses (0.03–0.05 mL) vertically within the deep fat compartment to increase eversion and projection, using one or two injection points depending on patient needs
WM	Mainly HYC‐24L (Juvéderm ULTRA XC) for its versatility in balancing definition, structure, and hydration of the lips in a natural manner	For accentuation of Cupid's bow/vermilion border, linear retrograde superficial subdermal injections (~0.05 mL per side) For upper lip eversion and projection, angled linear subdermal retroinjection in the tubercle; followed by similar injections slightly above the wet–dry border laterally for projection and hydration (varying from angled to horizontal as they proceed laterally depending on the degree of eversion needed); ~0.1 mL per side For lower lip projection, linear subdermal retroinjection in the middle third of the vermilion border (~0.02 mL); followed by linear subdermal retroinjections just below the wet–dry border for volume, projection, and hydration (~0.2–0.3 mL) For additional lower lip projection, linear, vertical, deeper subdermal retroinjections For hydration only, small, very superficial aliquots injected using a retrograde linear technique (both lips) in the superficial dry vermilion, with total volume < 0.5 mL
KMM	HYC‐24L (Juvéderm ULTRA XC) for its versatility in providing projection, eversion, and hydration HYC‐24L+ (Juvéderm ULTRA Plus XC) primarily in younger patients (particularly for use centrally)	For volumization, submucosal retrograde linear threads, not exceeding 0.1 mL, entering at the wet–dry transition and angling supero‐ or inferomedially towards the vermilion border For eversion, vertical linear submucosal threads, retroinjection of micro‐aliquots, spaced 1–2 mm apart, with rotation of the needle on exit For oral commissure support, an inverted‐K technique^a^

*Note:* Authors are listed in alphabetical order. Juvéderm ULTRA 2 and Juvéderm ULTRA XC can be considered as equivalent, as can Juvéderm ULTRA 3, Juvéderm ULTRA Smile, and Juvéderm ULTRA Plus XC.

^a^ Fine threads of product are placed inferior to the oral commissure, which supports and provides lift to the corner of the mouth.

The present authors usually use only one HA product per treatment session when injecting the lips. However, for experienced practitioners, it is possible to use two or more formulations in different areas or layers to provide synergistic benefits, either within the same or separate treatment sessions. For example, one of the group (KMM) sometimes uses both HYC‐24L (Juvéderm ULTRA XC) and HYC‐24L+ (Juvéderm ULTRA Plus XC) in younger patients wanting particularly full lips. In other instances, it may be beneficial to pair Hylacross products with HA gels from other product portfolios. Many of the present authors use VYC‐17.5L (Juvéderm VOLIFT/VOLLURE, Allergan Aesthetics, an AbbVie company, Pringy, Annecy, France) in some cases, for example when seeking extra projection or eversion in a mature lip. Furthermore, in patients with dry lips, some of the authors add an HA injectable with low G' and low cohesivity (for good spreadability) into the treatment plan to improve hydration—such as VYC‐12L (Juvéderm VOLITE/SKINVIVE, Allergan Aesthetics, an AbbVie company, Pringy, Annecy, France) [30, 31, 32].

### 2.5 Technical Considerations for Natural Outcomes With Hylacross Injectables

A number of different techniques have been published for treating the lips with HA injectables. Many of these were summarized in a recent review [33], and several other excellent approaches have since been added to the literature [26, 27, 34, 35]. In addition, the MD Codes system provides a systematic approach to HA‐based treatment of the face, including the lips [36], and may be particularly useful for less experienced practitioners.

Each of the present group has their own technical preferences—summarized in Table 4—and one of the authors (DB) has published his personal approach to lip treatment [25]. Importantly, despite varying techniques, there are some key commonalities. The authors would advise limiting administration to small volumes per injection point and, in most cases, suggest injecting no more than ~1 mL of product into the vermilion in a single session (Table 4). Volumetric planning should consider anatomico‐functional parameters, facial harmony, and the patient's esthetic objectives, with allowance for adjustment during follow‐up if needed. The majority of the authors favor the use of a needle rather than a cannula in most or all patients treated with Hylacross. Compared with cannulas, needles provide greater precision and control, allow for small deposits to be made into different lip compartments, and facilitate fine sculpting of delicate structures like the vermilion border and Cupid's bow. The smooth consistency of the Hylacross range may expedite this precise and controlled injection process. Nonetheless, it is possible to use a cannula instead, and some of the present authors consider that this device may be particularly appropriate in some complex cases, such as patients with elevated vascular risk (e.g., history of trauma, presence of scar tissue, or atypical vasculature). In addition, with aging lips, it may be necessary to address loss of perioral support prior to direct injection of the vermilion, and this can be achieved with fanning or linear threads delivered to the sub‐orbicularis oris fat pad—using a cannula to minimize the risk of injury to the labial arteries.

The achievement of natural outcomes is often crucial to patients, and Table 5 provides experience‐based technical considerations for attaining such results. An essential starting point is to assess the whole face and not just treat the lips in isolation. Particular attention should be given to adjacent structures in the perioral area, as well as bigonial width, cheek volume, chin width and length, nose width and projection, and nasolabial folds. This helps to ensure that the lips are properly framed and supported, leading to a balanced outcome. In addition, dental hygiene should be addressed prior to injection.

**TABLE 5:** Experience‐based considerations for achieving natural and well‐tolerated outcomes in the lips.

Consider the whole face and neck and not just the lips during treatment planning ○ Outcomes may be more balanced and proportionate if the lips are injected as part of a broader ‘full‐face’ approach rather than as isolated structures ○ Attention should be given to adjacent structures in the perioral area, as well as bigonial width, cheek volume, chin width and length, nose width and projection, and nasolabial folds Understand and respect natural lip anatomy and proportions ○ Consider the optimal upper‐to‐lower lip ratio appropriate to the individual patient (this may differ according to ethnic and cultural background) ○ Be aware of the highly compartmentalized structure of the lips ○ Established reference lines, such as the Ricketts E‐Line,^a^ may help to define treatment margins and prevent excessive projection beyond natural limits Assess baseline mucosal quality and ‘distensibility’ ○ In patients with small, dense, inelastic, or dehydrated lips, there may be a higher risk of irregularities and superficial product visibility Target the correct injection plane depending on the specific aims of treatment Take a conservative and multi‐staged initial approach ○ In most cases, avoid injecting more than ~1 mL into the vermilion in a single session ○ Consider adapting products volumes in real time during treatment, based on the dynamic response of the tissues ○ Use small volumes at each injection point Be wary of patients with unrealistic expectations or a desire for extreme results

^a^ The Ricketts E‐Line (Esthetic Line) is an imaginary line drawn from the tip of the nose to the tip of the chin.

In the authors' experience, it is also important to respect natural lip anatomy and proportions. The relative heights of the upper and lower lips may be particularly relevant; measured at the midline, a ratio between 1:1.6 and 1:2 is typically considered optimal in Caucasian females, although this will vary based on ethnicity and cultural preferences, and no single optimal ratio can be universally applied [15, 35]. In addition, practitioners should be mindful of the highly compartmentalized structure of the lips. A recent cadaver study identified 24 separate compartments, each of small size (0.30–0.52 mL) [13], which reinforces the notion that the volumes used at individual injection points should be small, in order to support natural expansion of each one. Practitioners may also wish to consider established reference lines—like the Ricketts E‐Line running from the tip of the nose to the tip of the chin [37]—in order to define treatment margins and prevent projection beyond natural limits.

Prior to injection, it may also be worthwhile to assess baseline mucosal quality and “distensibility” to ensure that the underlying anatomy is favorable for augmentation, particularly in patients wanting significant volume enhancement. Among individuals with small, dense, inelastic, or dehydrated lips, there may be a higher risk of irregularities and superficial product visibility.

In addition, selection of the correct injection plane is very important, depending on the specific aims in a given treatment area. Deeper deposits may be best for structural support and projection, while superficial injections are more appropriate for definition and refined contouring. Appropriate selection of injection plane—as well as the avoidance of excessive treatment volumes—can also help to reduce the risk of product displacement, which is important in ensuring natural outcomes. In the authors' experience, the likelihood of displacement may be increased if channels are made between the cutaneous lip and vermilion, so it is advisable to avoid injection techniques that breach the vermilion border, particularly in the upper lip.

In the authors' experience, it is generally advisable to take an initially conservative and staged approach to lip improvement with treatment‐naïve patients. By starting with small overall volumes and reassessing the need for more at subsequent visits, optimal outcomes can be built up over time, and the risk of overfilling is greatly reduced. Furthermore, it is particularly important when treating the lips not to be dogmatic about treatment volumes; dosing should always be individualized in the planning phase and can also be re‐evaluated in real time during treatment itself (e.g., based on the behavior of the mucosa and surrounding structures).

It is essential to evaluate the results of lip treatment over time. Standardized photographic documentation at baseline, immediately post‐injection, and then throughout follow‐up facilitates accurate assessment. To achieve this, photographs should be taken using consistent lighting and background, and from the same angles each time, ideally both at rest and during animation (e.g., smiling) [38]. The patient's perioral area should be free from make‐up. If required, objective assessments can be made using validated instruments like the Lip Fullness Scale [39]. Patient satisfaction should also be monitored. This can be done informally, although validated, standardized tools are also available, such as FACE‐Q [40].

**FIGURE 1:**
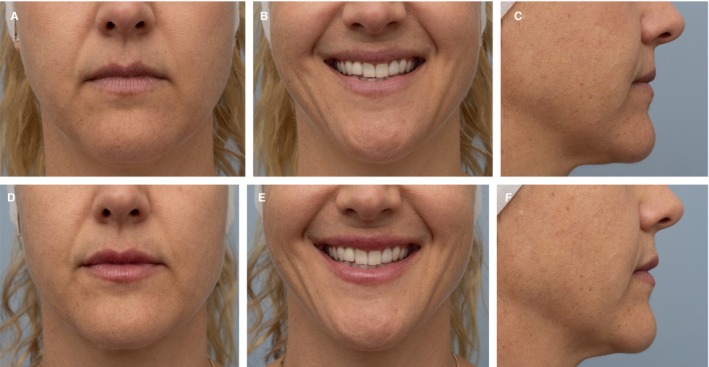
Lip improvement using Hylacross injectables. This 40‐year‐old female was treated using 0.5 mL of HYC‐24L (Juvéderm ULTRA XC) injected subdermally into the upper and lower vermilion bodies using a needle. She is shown before (A–C) and 4 weeks after treatment (D–F). Treatment of the lips produced subtle volume and eversion, improved hydration, and enhanced coloration of the vermilion. Images are courtesy of author SGB.

**FIGURE 2:**
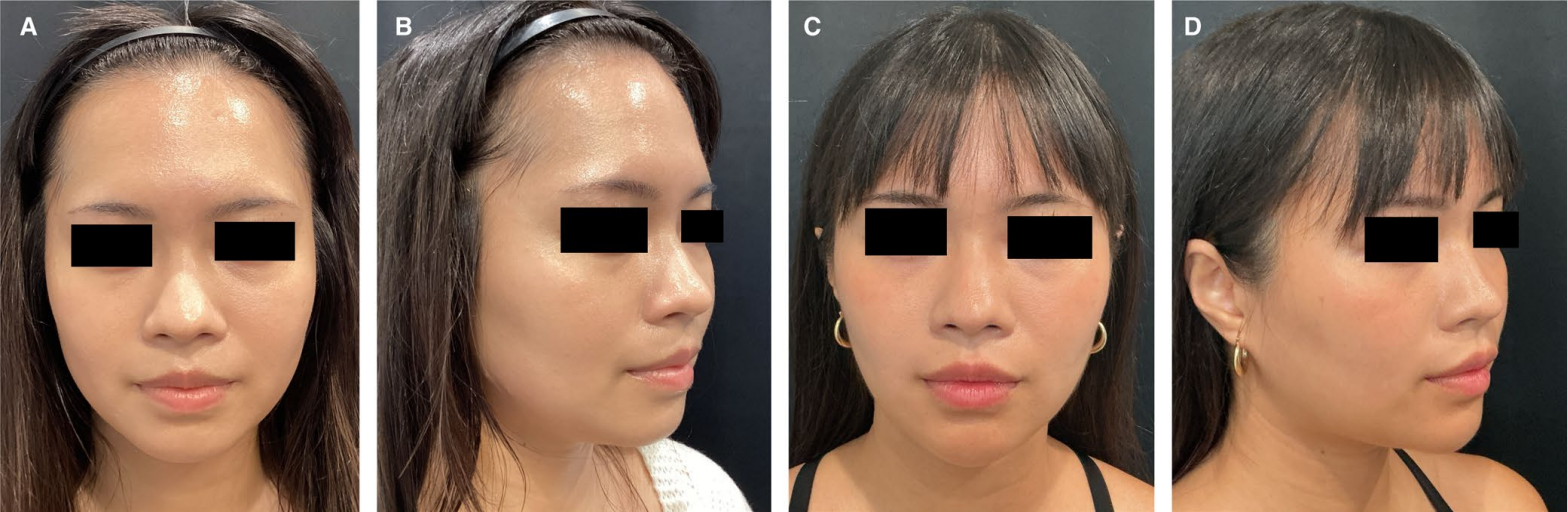
Lip improvement using Hylacross injectables. This 27‐year‐old female was treated using 1 mL of HYC‐24L (Juvéderm ULTRA XC) injected subcutaneously using a needle, based on linear threads and vertical micro‐threads. She is shown before (A and B) and 6 months after treatment (C and D). The impact of treatment is evident in subtle volumization and eversion. The patient was also treated using 3 mL of VYC‐20L in the cheeks and chin and 2 mL of VYC‐25L in the jawline. Images are courtesy of author KMM.

**FIGURE 3:**
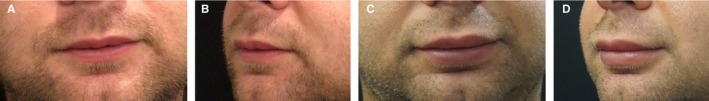
Lip improvement using Hylacross injectables. This 29‐year‐old male was treated using 1 mL of HYC‐24L (Juvéderm ULTRA XC) and then 3 weeks later using 1 mL of HYC‐24L+ (Juvéderm ULTRA Plus XC). Both rounds of injections were made subcutaneously using a needle, based on a linear threading technique. He received no other esthetic treatments. The patient is shown before (A and B) and 3 months after initial treatment (C and D), and the impact of treatment is particularly evident in the subtle volumization achieved. Images are courtesy of author KMM.

**FIGURE 4:**
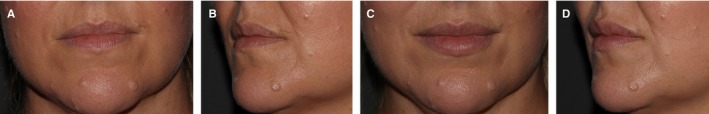
Lip improvement using Hylacross injectables. This 43‐year‐old female was treated using 0.8 mL of HYC‐24L (Juvéderm ULTRA XC) injected using a needle. The upper vermilion border and the middle of the lower vermilion border were injected into the superficial subdermal plane using a linear technique for greater definition. In addition, the body of both lips was injected into the immediate subdermal plane using a linear technique to give more volume and hydration, and the lower lip tubercles were treated with a vertical injection into a deeper subdermal plane. She is shown before (A and B) and 1 month after treatment (C and D). Images are courtesy of author WM.

**FIGURE 5:**
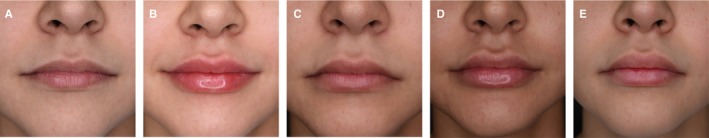
Lip improvement with repeated treatment using Hylacross injectables. This 20‐year‐old female was treated over two sessions to promote integration, minimize the risk of irregularities, and achieve a harmonious result. Initially, she received 0.7 mL of HYC‐24L+ (Juvéderm ULTRA Plus XC) injected using a needle. The product was administered into the subdermal plane of the lower vermilion border for definition; into the subdermal plane or superficial fat compartment of the upper lip for verticalization and mucosal hydration; and into the deep fat compartment/submucosal plane for eversion and volume. She is shown before treatment (A), immediately afterwards (B) and at 9 months (C). The patient was then re‐treated based on similar techniques using 0.6 mL of HYC‐24L+ (Juvéderm ULTRA Plus XC). She is shown immediately after the second treatment (D) and then at 9 months (E). Images are courtesy of author LMCO.

Figures 1, 2, 3, 4, 5 show examples of patients who have achieved natural outcomes following lip treatment using Hylacross products. All provided written informed consent for use of their images.

### 2.6 Minimizing Risk With Hylacross Injectables in the Lips

As with any HA injectables, there are certain key steps that practitioners are strongly advised to undertake prior to treating the lips with Hylacross products to ensure safer outcomes. In particular, they should develop expert knowledge of lip anatomy, seek professional training in relevant injection techniques, and understand the physicochemical and rheological properties of each product they intend to use.

Prior to treatment, a detailed review of the patient's medical history should be performed, including previous procedures in the perioral area, and any possible risk factors for poor tissue quality or compromised healing—such as anticoagulant use or smoking. In the authors' experience, potential contraindications specific to lip treatment include active labial herpes simplex virus infection, significant perioral acne or dermatitis, and poor dental hygiene.

From a practical perspective, many of the previous considerations relating to natural outcomes (Table 5) are also pertinent to patient safety. In addition, before starting to inject, it is advisable to assess the position and depth of the labial arteries as far as possible in order to minimize the risk of vascular compromise during administration. Careful observation at rest and in motion, combined with gentle palpation, can help to identify vascular trajectories; ultrasound may also be used if preferred. This is particularly important given that both the superior and inferior labial arteries show high levels of inter‐individual variability in their path and depth [41].

Other key safety principles include aseptic non‐touch technique (e.g., thorough cleaning of the treatment area, use of gloves, and avoidance of touching the injection device), slow and controlled product administration, use of aspiration, and careful monitoring for signs of vascular compromise (e.g., unexpected pain, blanching, or livedo) [38]. Rinsing the patient's mouth with antiseptic mouthwash prior to injection may also be a useful preventive measure [38]. When using a cannula, it is best to avoid gliding on the skin before penetration as this can collect bacteria. In addition, if molding the product from inside the lip, it is advisable to use a gauze or change gloves before continuing with injection. Practitioners are also encouraged to familiarize themselves with potential complications and their management. In the unlikely event of emergency, established protocols must be in place for immediate intervention, including a ready supply of hyaluronidase and any other relevant remedies.

Finally, patients should be given simple after‐care instructions, such as avoiding make‐up, excessive touching of the lips, or strenuous exercise for 24–48 h. They must also be advised to contact the clinic immediately if they experience pain, excessive swelling, or color changes in the treatment area [38].

### 2.7 Managing Patient Concerns

For many patients, a key anxiety around HA‐based lip treatment is fear of unnatural results—for example, so‐called “duck lips” or “trout pout”. This issue is not specific to the Hylacross range or any other brand, but it may lead some individuals to reject HA injectables despite wanting esthetic improvement of the lips. In the authors' experience, it is often possible to overcome such fears with thoughtful patient management. This requires a comprehensive approach that combines relationship building, patient education, and conservative treatment planning.

The first step is to build trust. To do this, clinicians should listen with empathy to the patient's esthetic aspirations and desires. Emotional support is also key. Their worries are often not just physical but also psychological—of not recognizing themself in the mirror or facing social judgment from others. It is important to emphasize to patients that the clinical goal is to enhance appearance while respecting personal identity.

The second step should be personalized patient education. With the Hylacross range, this can include explanation of the specific characteristics underlying product selection, their ability to integrate naturally into the tissue, and their long track record of effective, durable, and well‐tolerated outcomes. Clinical study data can be shared if the patient is interested. Discussion of the potential to reverse the procedure with hyaluronidase may also help to reassure the patient. Additional peace of mind can be provided by sharing before‐and‐after photographs of individuals who have recently been treated by the same practitioner and informing the patient how many previous lip procedures they have performed with the chosen product. Moreover, it is important to clarify that unnatural outcomes are usually the result of overfilling and/or poor technique rather than being product‐related—and therefore can be avoided through good clinical practice.

A third key step that is particularly important with hesitant patients is to take a conservative and multi‐staged approach to treatment, building up the result over several sessions. As already described, this reduces the potential for overfilling and also decreases the likelihood of an excessively large change in appearance based on a single session, which can be stigmatizing. The hygroscopic nature of Hylacross products may give a more volumized look in the first few weeks, which then stabilizes over time, making it even more important to take a staged approach. It can also be reassuring to pause occasionally during the treatment to allow the patient to see current progress in the mirror and discuss any potential concerns.

Finally, practitioners should always forewarn patients about common and normal short‐term adverse events of HA injections in the lips, such as mild local swelling, bruising, and tenderness. They are less likely to worry unduly if they are properly prepared.

### 2.8 Limitations and Future Work

The present paper synthesizes the knowledge and experience of using Hylacross injectables in the lips among a global group of expert practitioners. Nonetheless, the authors acknowledge that there are limitations. In particular, the underlying trial data are now more than 10 years old [9, 10, 11]. Bridging the gap to contemporary practice was a key rationale for developing the present paper but readers are nonetheless encouraged to enrich the literature by publishing results from their own practices. There is also a need to further assess the potential of combined approaches to lip improvement based on more than one HA formulation and/or co‐treatment with other modalities, and to examine long‐term safety and effectiveness across multiple treatment sessions. In addition, the authors acknowledge that while their personal preference when treating the lips is often for Hylacross injectables, other practitioners will have their own experiences, techniques, and product preferences for achieving their patients' goals.

## 3 Conclusions

HA injectables can be used for lip improvement across a wide range of patient needs, such as countering age‐related changes, volume augmentation, improving symmetry and balance, contouring, and hydration. The Hylacross portfolio of products has rheological and physicochemical properties that make them well suited to lip enhancement, and they are supported by robust data from clinical trials and a wealth of experience in daily practice. Most patients want natural results from lip treatment, and achieving this requires adherence to key technical principles—including respect for individual lip anatomy and proportions, careful selection of injection device and depth, and the use of product volumes that are appropriate to the treatment goals and patient anatomy.

## Author Contributions

C.G. designed the questionnaire. S.G.B., K.M.M., L.M.C.O., W.M., H.A., D.B., and C.G. contributed to the conception and design of the paper and the interpretations provided; S.G.B., K.M.M., L.M.C.O., W.M., H.A., D.B., and C.G. were involved in drafting the manuscript and revising it critically for important intellectual content; S.G.B., K.M.M., L.M.C.O., W.M., H.A., D.B., and C.G. gave final approval of the version to be published; and S.G.B., K.M.M., L.M.C.O., W.M., H.A., D.B., and C.G. agreed to be accountable for all aspects of the work.

## Funding

Writing and editorial assistance was provided to the authors by Dr. Timothy Ryder from Biological Communications Limited (London, United Kingdom) and funded by Allergan Aesthetics, an AbbVie company. Neither honoraria nor payments were made for authorship.

## Ethics Statement

The authors have nothing to report.

## Consent

All of the patients whose photographs are used in this publication provided written informed consent.

## Conflicts of Interest

Sarah G. Boxley is a consultant and investigator for Allergan Aesthetics, an AbbVie company. Kathleen M. Morno is a speaker and consultant for Allergan Aesthetics, an AbbVie company. Lina María Cadena Ortiz is a speaker for Allergan Aesthetics, an AbbVie company. William McGillivray is a speaker, consultant, and investigator for Allergan Aesthetics, an AbbVie company. Hani Aljohani reports nothing to disclose. Dario Bertossi is a speaker and consultant for Allergan Aesthetics, an AbbVie company. Carola de la Guardia is an employee of Allergan Aesthetics, an AbbVie company.

## Supporting information


**Data S1:** Questionnaire: Experience with HYC‐24 in lips.

## Data Availability

AbbVie is committed to responsible data sharing regarding the clinical trials we sponsor. This includes access to anonymized, individual, and trial‐level data (analysis data sets), as well as other information (e.g., protocols, clinical study reports, synopses, or statistical analysis plans [SAPs]), as long as the trials are not part of an ongoing or planned regulatory submission. These clinical trial data can be requested by any qualified researchers who engage in rigorous, independent, scientific research, and will be provided following review and approval of a research proposal, SAP, and execution of a data use agreement (DUA). Data requests can be submitted at any time after approval in the US and Europe and after acceptance of this manuscript for publication. The data will be accessible for 12 months, with possible extensions considered. For more information on the process or to submit a request, visit the following link: https://vivli.org/ourmember/abbvie/ then select “Home”.
