# Supplementary material for: Hylacross Hyaluronic Acid Injectables in the Lips: Global Expert Perspectives for Achieving Optimal Esthetic Outcomes
Source: J Cosmet Dermatol. 2026 Jul 3;25(7):e71013. doi: 10.1111/jocd.71013 (PMC13332343; doi:10.1111/jocd.71013)
Supplement: Supplementary file 1 — Data S1: Questionnaire: Experience with HYC‐24 in lips. [file JOCD-25-e71013-s001.docx]

**Questionnaire: Experience with HYC-24 in lips**

**Thank you for participating in this expert opinion paper about treatment of the lips with Juvéderm ULTRA fillers (HYC-24). We would be grateful if you could complete the following questionnaire.**

******For this manuscript, please note that “LIPS” includes the lip body (vermillion) and its border but not the perioral area (ie, it does not include upper lip lines, barcode lines, oral commissure lines, or marionette lines).***

**Question 1.** When did you start practicing in aesthetic medicine? *[please give a year]*

|  |
| --- |

**Question 2.** When did you start using Juvéderm ULTRA fillers? Which ones do you use? These include: Juvéderm ULTRA, Juvéderm ULTRA XC, Juvéderm ULTRA PLUS, Juvéderm ULTRA PLUS XC, Juvéderm ULTRA 2, Juvéderm ULTRA 3, Juvéderm ULTRA SMILE, Juvéderm ULTRA 4. *[please give a year and the products you use]*

|  |
| --- |

**Question 3.** Approximately how many individual patients have you treated with Juvéderm ULTRA fillers (for any facial area) in all these years? *[if you use several products, please provide an estimate with each one]*

|  |
| --- |

**Question 4.** Specifically for treating the **lips**, what are the available nonsurgical medical aesthetic treatment modalities, and which ones do you use?

|  |
| --- |

**Question 5.** When you treat the **lips**, do you usually inject one HA product or do you use a combination of products? *[please list all of the products and combinations you use, including non-Juvéderm products, and the reasons for using each one]*

|  |
| --- |

**Question 6.** Approximately how many individual patients have you treated with Juvéderm ULTRA fillers in the **lips**? *[if you use several Juvéderm ULTRA* *products, please provide an estimate with each product for the lips]*

|  |
| --- |

**Question 7.** Approximately how many individual patients have you treated repeatedly with Juvéderm ULTRA fillers in the **lips**? *[i.e. treated at least 3 times with Juvéderm ULTRA fillers]*

|  |
| --- |

**Question 8.** What are your typical patient profiles (characteristics) for treating the **lips** with Juvéderm ULTRA fillers?

|  |
| --- |

**Question 9.** What are the unique differentiating characteristics of Juvéderm ULTRA fillers for treating the **lips**? *[i.e. what are your reasons for using Juvéderm ULTRA fillers in the lips; please list at least three reasons]*

|  |
| --- |

**Question 10.** Please explain how you inject the lips with Juvéderm ULTRA fillers and the reasons for your technique *[i.e. needle/cannula, depth of injection, volumes, etc]*

|  |
| --- |

**Question 11.** How do you assess the outcomes in lips and how do you record it in the patient´s file? *[i.e. do you use any scales, imaging, satisfaction scales, etc]*

|  |
| --- |

**Question 12.** Based on your experience, what would be your advice to other injectors to ensure natural outcomes when using Juvéderm ULTRA fillers in the **lips**?

|  |
| --- |

**Question 13.** Based on your experience, what would be your advice to other injectors to ensure safe outcomes when using Juvéderm ULTRA fillers in the **lips**?

|  |
| --- |

**Question 14.** Based on your experience, what would be your advice to other injectors for overcoming their patients’ fears about using Juvéderm ULTRA fillers in the **lips**?

|  |
| --- |

Add any other comments that you feel are relevant for treating the lips with Juvéderm ULTRA fillers

|  |
| --- |
